# “Global Health”: Time to Refocus while We still Have Time

**DOI:** 10.4269/ajtmh.20-0081

**Published:** 2020-04-17

**Authors:** David Bell, Eliah Aronoff-Spencer

**Affiliations:** 1 Independent Consultant, Issaquah, Washington;; 2 Infectious Diseases and Global Public Health, University of California San Diego, San Diego, California

## Abstract

Two decades of growing resource availability from agencies and foundations in wealthy countries has transformed approaches to health in poorly resourced nations. This progress looks increasingly unstable as climate change, social unrest, and, now, disruptive pandemics present threats not only to health but also to the mechanisms that manage it, and to funding itself. The growth in “global health” schools, technology development laboratories, nongovernmental organizations and multilateral institutions in donor countries has delivered not only successes but also disappointment, and reflect a paradigm that is in many ways contrary to the principles of population-based ownership that they espouse. Although the COVID-19 crisis has underlined the importance of health access and health service capacity, we may have a limited window of opportunity in which to rethink the current model and improve both efficiency and effectiveness. With a dose of humility, we may all benefit from studying our own rhetoric on human-centered design and applying these principles across global health to ensure that our approach is effective, efficient, and defensible.

The term “Global Health” has little immediate relevance to most Americans, distracted by fires, pandemics, or questioning the country”s very character and relationship with the wider world. Rather, “Global Health” evokes a model in which wealthy countries describe and address the health, or lack thereof, of those less well-off—people in countries that most are unlikely to travel to, that are recipients of Western “largesse.” The “Global Health community,” despite perhaps feeling generally above this, is not clearly different, often skating on the edge of a colonial mindset of “us” and “them.” This approach fails the test of logic in terms of a truly global approach and misses the point by lumping together the health of “poor” populations—thereby failing to acknowledge the heterogeneity that defines both the problem and the required response.

The current novel coronavirus disease (COVID-19) pandemic underlines the interconnectedness of health globally and the risks to all of us of maintaining inequalities in healthcare capacity and infrastructure (no country is a health island anymore). Accelerating climate change, civil unrest and resultant migration are set to make diseases previously lumped into this “global health” basket increasingly global. It should now be clear (post-COVID-19 spread) that global health should truly be global and concern us all. But the burden and the capacity gap remain focused in low-resourced populations. No one is immune to current and coming global crises; it therefore seems imperative to review the effectiveness, efficiency, and equality of our current approaches in the face of these emerging threats, competing priorities, and potentially declining resources. Even after COVID-19, it is unclear whether a likely boost in preparedness funding will address this gap or be solely focused on protecting “our own” populations (even the European Union has clear internal borders again).

With the surge in funding in recent decades for HIV/AIDs, tuberculosis, and malaria in particular, from both public agencies and private philanthropies, Global Health has grown into a significant industry with proliferating university based “Global Health” schools and expanding nongovernmental organizations (NGOs) specializing in managing and deploying resources. These institutions are centered in wealthy countries, strategically close to funding sources and sharing their same culture—an illogical situation from the point of view of the problem they are seeking to address, and perhaps a systematic error that retards the development of the innovative and sustainable global healthcare (substitute “civil engineering” and “climate stewardship”) we really need. While recognizing that some large Western-based NGOs have strong regional and country offices, advances in communication technologies should mean that the previous imperative for geographical proximity in communication with funding sources no longer applies. However, it is noted that change would require confidence that the funding sources are also willing to deal with a leadership of recipient organizations that is geographically remote.

The rise of funding mechanisms in the last two decades has doubtlessly contributed to the global reduction in diseases of poverty, complementing an underlying general economic improvement of most low-income populations. However, while the HIV epidemic (that impacted the rich along with those less wealthy) has been stemmed—it is far from over,^1^ progress on TB is well below target,^2^ and malaria’s decline has stalled and is possibly in reverse.^3^ Worse, emerging pathogens including Ebola and Zika are exacerbated by this confluence of climate change, and/or inadequate civil infrastructure and local capacity. Like a hub and distributed spoke model, the key funded responders are tasked to address the problems of populations with whom they often have little geographical connection or lived expertise. This approach, hearkening to roots in the industrial revolution, flies in the face of modern innovation theory. Although external subject matter expertise remains vital in all geographies and fields, context is also essential, problems are best solved in the places they most frequently occur, with populations facing them every day.^4^

Recent investment is not without successes. Drug and vaccine development, requiring years of highly specialized purely laboratory-based research, have borne great fruit. Pneumococcal, HiB, meningococcal, RotaV vaccines and antiretroviral therapies are transforming the lives and outlook of millions. However, the disconnect between priority-setters and their target populations becomes apparent in the delivery of technology at the last mile: devices, diagnostics, and software applications aimed at improving the day-to-day functioning of community-level health systems, where the keys to most avoidable mortality reside. These fields are crowded with good intentions, but decades after Alma-Ata, “primary care” is still frequently viewed by funders as a niche interest for operational research, not as a well-resourced fundamental norm. Although the disease-focused funding of the Global Fund, the largest disbursement mechanism for global health, have provided broader benefits, a small fraction of expenditure in health systems strengthening is outside the single-disease–focused programs—$67 million of 3.9 billion dispersed in 2018.^5^

The management of acute fever in children, a pillar of the WHO strategy, is a good exemplar of missing the forest for the trees. Although the presenting symptom in most deadly yet preventable childhood illness, fever is inconvenient to package, being of diverse etiology and demanding accordingly varied management. This presents a problem too hard (too boring?) to manage from a distance or summarize in sound bites. Rather, the approach of the global health community has been one that an outsider might adopt—to pick a disease that is more readily packaged, malaria, and develop vertical programs to target this significant, but not predominant, cause of mortality. Reducing and eliminating malaria has become central to global health planning from the G8 down and remains a major driver of investment. It has probably accelerated local elimination on the periphery of global transmission, but with the decline in burden stalling or reversing in the last few years, it behooves us to look hard at why this is so.^3^ A concerted and more locally driven clinical and public health approach, understanding the social and environmental determinants of disease, could achieve a significantly broader impact with lower overall cost while building the infrastructure and knowledge capacity that true sustainability demands.

The call to eliminate malaria came from wealthy countries—it was not a mass movement from those who live within its immediate reach. No mother wants her child to die of malaria or from pneumonia, diarrhea, meningitis, or other states associated with poverty, malnutrition, and under-resourced health systems. As elimination approaches, malaria holds even less significance to the daily lives of those living with it. People know what their own priorities are. They also have knowledge of local climate, logistical challenges, and customs. It could reasonably be argued that they are in a better position to prioritize not only on implementation but also on health priority setting. Impressive successes in malaria reduction, such as in eastern Myanmar and Sri Lanka, are notable for being locally driven and show the impact of strong community engagement.^6–8^

We are still living within the bubble of relative largesse in global health funding. Private philanthropy remains powerful in bringing attention and in driving priorities, and generous public spending persists, although wavering in the face of populist (and perhaps often justified) skepticism. It would be exciting to envision what might happen if the center of gravity for directing those funds could cross the divide toward the center of preventable mortality—if investment in “Global Health” schools could go to the University of Zambia rather than the University of Washington, or of Lagos rather than London. Specialist learning would become more accessible to those dealing with these problems day to day. Greater local context could reduce the inefficiencies of externally imposed programs and deadlines. Perhaps, in this postcolonial world, a re-headquartering of the WHO in Addis Ababa or Delhi would bring the immediacy and urgency in priority setting to the persisting epidemics of malaria, TB, and maternal and infant mortality that we have seen with the European outbreaks of COVID19. Would the Delhi smog be tolerated if the WHO staff breathed it daily? The industry grown around aid within wealthy countries would suffer, but that window may be closing anyway.

Concentrating capacity in low- and middle-income countries with their attractive wage and costs structures, and where most of the global population actually resides, will build local economies, and thereby perhaps offer an exit strategy for aid-weary taxpayers. The clustering of health NGOs and consultants around organizations such as the WHO could pay real dividends in these environments. The presence of technical expertise would facilitate local capacity building and may help reverse current brain drains. A greater sense of local and regional ownership may stimulate greater contribution from low-income nations to multilateral funds such as the Global Fund or GAVI, and new models for dispersal of this funding. Examples such as the Wadhwani Initiative for Sustainable Healthcare Foundation in India or BRACS in Bangladesh illustrate the impact of local knowledge and control.^9,10^ PEPFAR’s community grant programs and the long-term support of in-country research centers by the Wellcome Trust, the European European & Developing Countries Clinical Trials Partnership program, and U.S. NIH provide encouraging examples of how required capacity building could be attained, and that such direct linkages can be effective and sustained.

In other contexts, the spending of funds intended to benefit low-income countries on high-wage workers in rich economies and concentrating training on those with the least relevant background would be considered scandalous. The enormous budget that sustains the transport of wealthy experts across the globe, funds research in expensive laboratories, and gathers leaders to share insights in often luxurious settings points to not just a mistake in strategy, and perhaps morality, but a fundamental economic misadventure. Although COVID-19, Ebola, HIV, and climate projections all underline the importance of having strong coordinated approaches, maybe it is time for a global experiment, moving the center of gravity in Global Health from Lake Geneva to Lake Taganyika. In an increasingly unstable world, we may have limited time to change.

## References

[1] GBD 2017 HIV collaborators, 2019 Global, regional, and national incidence, prevalence, and mortality of HIV, 1980–2017, and forecasts to 2030, for 195 countries and territories: a systematic analysis for the Global Burden of Diseases, Injuries, and Risk Factors Study 2017. Lancet HIV 6: e831–e859. 31439534 10.1016/S2352-3018(19)30196-1 PMC6934077

[2] WHO, 2019 Global Tuberculosis Report 2019. Geneva, Switzerland: World Health Organization.

[3] WHO, 2019 World Malaria Report 2019. Geneva, Switzerland: World Health Organization.

[4] Baldwin C von Hippel E, 2011 Modeling a paradigm shift: from producer innovation to user and open collaborative innovation. Organ Sci 22: 1399–1417.

[5] The Global Fund 2018 Annual Financial Report. 2019. The Global Fund to Fight AIDS, Tuberculosis and Malaria. Geneva. Switzerland Available at: https://www.theglobalfund.org/media/8470/corporate_2018annualfinancial_report_en.pdf?u=637146788010000000. Accessed March 18, 2020.

[6] Premaratne R Wickremasinghe R Ranaweera D Kumudu WM Gunasekera TdeAW Hevawitharana M Pieris L Fernando D Mendis K, 2019 Technical and operational underpinnings of malaria elimination from Sri Lanka. Malar J. 18: 256. 31358007 10.1186/s12936-019-2886-8 PMC6664748

[7] Parker DM Landier J Thu AM Lwin KM Delmas G Nosten FH, Malaria Elimination Task Force Group, 2017 Scale up of a *Plasmodium falciparum* elimination program and surveillance system in Kayin state, Myanmar. Wellcome Open Res. 22: 98. 10.12688/wellcomeopenres.12741.2 PMC5701446 29384151

[8] McLean ARD 2018 Malaria elimination in remote communities requires integration of malaria control activities into general health care: an observational study and interrupted time series analysis in Myanmar. BMC Med 16: 183. 30343666 10.1186/s12916-018-1172-x PMC6196466

[9] WISH, 2020 Wadhwani Initiative for Sustainable Healthcare (WISH). Available at: https://www.wishfoundationindia.org/. Accessed January 21, 2020.

[10] BRAC, 2020 BRAC Bangladesh. Available at: http://www.brac.net/. Accessed January 21, 2020.

